# Application of artificial intelligence tools and clinical documentation burden: a systematic review and meta-analysis

**DOI:** 10.1186/s12911-025-03324-w

**Published:** 2025-12-24

**Authors:** Jungang Zhao, Hanxiang Liu, Yaolong Chen, Fujian Song

**Affiliations:** 1 Department of Pediatric Research Institute, National Clinical Research Center for Children and Adolescents’ Health and Diseases, Ministry of Education Key Laboratory of Child Development and Disorders, Chongqing Key Laboratory of Child Rare Diseases in Infection and Immunity, Children’s Hospital of Chongqing Medical University, Chongqing, China; 2 Chevidence Lab of Child & Adolescent Health, Children’s Hospital of Chongqing Medical University, Chongqing, China; 3 Norwich Medical School, University of East Anglia, Norwich, Norfolk, UK; 4 Research Unit of Evidence-Based Evaluation and Guidelines, School of Basic Medical Sciences, Chinese Academy of Medical Sciences (2021RU017), Lanzhou University, Lanzhou, China; 5 WHO Collaborating Center for Guideline Implementation and Knowledge Translation, Lanzhou, China

**Keywords:** Artificial Intelligence, Clinical Documentation, Large Language Models, Burnout, Workload, Healthcare workers

## Abstract

**Background:**

Clinician burnout is a growing global concern, with heavy clinical documentation workload identified as a major contributor. Clinical documentation tasks, though essential for patient care and communication, are time-consuming and cognitively demanding. Recent advances in artificial intelligence (AI), particularly natural language processing and large language models, are being explored as potential tools to alleviate documentation burden, yet their quantitative impact has not been systematically assessed.

**Methods:**

We performed a systematic review and meta-analysis, registered on PROSPERO (CRD420250653291) and guided by PRISMA. Eligible studies included frontline health professionals using AI tools for clinical note creation, with comparators being usual practice or pre-implementation baseline. Primary outcomes were documentation burden, workload, burnout, and time spent on documentation. Searches were conducted in PubMed, Web of Science, Scopus, and key journals. Effect sizes were synthesized using standardized mean difference (SMD) under a random-effects model, with subgroup analyses by study design, AI tool type, task type, editing status, and data origin.

**Results:**

Of the 23 studies included, 12 were non-randomised studies with a concurrent control and 11 employed a before-and-after comparison design. The study participants varied in specialties and were mainly from ambulatory settings, including physicians, surgeons, pediatricians, and ICU specialists. Heterogeneity in results across included studies was considerable, and the methodological quality of the available studies was generally low. Pooling results of the 14 studies yielded an overall standardized mean difference (SMD) of -0.71 (95% confidence interval [CI]: -0.93 to -0.49), indicating a moderate reduction in documentation workload and related burnout. Based on results of studies in which clinicians reviewed and edited AI-generated drafts, AI applications reduced documentation time, similarly representing a moderate effect size (SMD= -0.72, 95% CI -0.99 to -0.45). The quality of notes generated by AI tools was at least comparable to those prepared manually by clinicians.

**Conclusions:**

AI technologies offer promising benefits for reducing clinical documentation burden. However, their implementation must be accompanied by rigorous quality control and ongoing evaluation in practical settings to optimize their effectiveness and safeguard patient care outcomes.

**Supplementary Information:**

The online version contains supplementary material available at 10.1186/s12911-025-03324-w.

## Introduction

High emotional exhaustion and symptom of burnout were found among healthcare providers globally, for instance, among 39% and 44% of US physicians [1]. The heavy documentation workload is a significant contributor to clinician burnout [2]. Clinical documentation refers to the comprehensive recording of a patient’s medical history, treatment plans, diagnoses, test results, and other pertinent healthcare information. These records are essential for ensuring continuity of care, facilitating communication among healthcare providers, and supporting clinical decision-making. Examples of clinical documentation include clinical notes following a clinician-patient encounter, admission notes, discharge summaries, operative reports, and response notes to messages from patients.

The time-consuming nature of clinical documentation may detract clinicians’ attention from direct patient care, leading to decreased job satisfaction and increased stress among healthcare workers. Documentation tasks often require meticulous attention to detail and can be repetitive and tedious, exacerbating feelings of cognitive exhaustion and burnout among clinicians. This chronic stress not only affects the mental and physical health of clinicians but also impacts the overall quality of care provided to patients [3]. Addressing the clinical documentation burden is crucial for mitigating burnout and improving healthcare outcomes [2]. 

The documentation burden is exacerbated by the inefficiency of current electronic health records (EHRs) interfaces and regulatory coding requirements. Studies estimated that clinicians spend nearly two hours on EHR tasks for every hour of direct patient care, often forcing them to complete documentation tasks outside normal working hours—a phenomenon widely known as ‘pajama time’ [4, 5].

Recent advance in artificial intelligence (AI), natural language processing (NLP), and large language model (LLM) technologies has been increasingly integrated into healthcare processes. Current applications have evolved beyond simple speech-to-text dictation. Emerging ‘Ambient Clinical Intelligence’ (ACI) tools can now autonomously draft structured clinical notes by listening to patient-clinician conversations [6], while other generative AI models are being deployed to draft responses to the surging volume of patient inbox messages [7]. Specifically, it has been hoped that the use of AI and LLM tools may reduce the documentation burden faced by healthcare workers [8], although some are not so convinced [9, 10]. There were several published scoping reviews or systematic reviews regarding AI applications on clinical documentation [11–14]. The existing reviews were narrative in nature and not focused on documentation burden related outcomes. Our meta-analysis aimed to quantitatively synthesize data from published studies that evaluated the effects of AI applications for clinical note creation on the documentation burden, time expenditure, and related outcomes.

## Methods

The protocol for this meta-analysis is registered on PROSPERO (CRD420250653291). The report of this systematic review was guided by the Preferred Reporting Items for Systematic Reviews and meta-analyses [15]. 

### Eligibility criteria

Studies were included in this meta-analysis if they meet the following criteria:

- *Participants*: Front-line health professionals, including physicians, nurses, and allied health professionals (e.g., physical therapists, occupational therapists, and speech-language pathologists).
- *Interventions*: The use of AI technologies for the creation of clinical notes, including the preparation of documentation entries within electronic health records, discharge summaries, and letters to patients.
- *Outcomes*: The primary outcomes are documentation burdens, workload, cognitive burnout or emotional exhaustion, and time spent on documentation tasks. Secondary outcomes included accuracy (errors) and quality of AI-generated clinical notes and documents.
- *Control/comparisons*: Usual practice before or without the application of AI tools.
- Study designs: Studies that concurrently compared the application of AI-based tools with a control system, or studies that compared changes in documentation burden before and after implementing AI technologies.
- We included studies published in journals or as preprints written in English.

We excluded studies without providing data on documentation burden or time spent on documentation. We also excluded studies that evaluated AI applications for reviewing medical records only, or making diagnostic recommendations, or clinical decision support system without a focus on note creation.

### Data sources

We searched PubMed, Web of Science, and Scopus to identify relevant studies, using keywords and phrases related to artificial intelligence or AI, large language models (LLM), and documentation burden or workload of healthcare professionals (see Supplementary file-1 for PubMed search strategy). In addition, we manually checked the references of retrieved articles and conducted forward and backward citation chaining of relevant studies. We also searched contents of the following journals: NEJM AI, NEJM Catalyst Innovations in Care Delivery, JAMIA Open, and JMIR AI. Given the rapid evolution of AI and LLM technologies, we included preprints to capture the most recent evidence and minimize publication bias. The quality of these studies was rigorously assessed using the same tools applied to peer-reviewed articles.

### Study selection

Identified records from all databases were downloaded, managed, and de-duplicated using EndNote. We then used the Automatic Systematic Reviews (ASReview), a half-automatic machine-learning (ML) platform [16] to prioritize records for title and abstract screening. After receiving prior knowledge (including a few relevant and irrelevant studies already known), ASReview algorithms applied natural language processing to automatically rank the remaining studies according to their predicted eligibility. Then a researcher decided whether the study ranked first is relevant or irrelevant. Based on the researcher’s last decision, ASReview re-ranked unscreened studies by newly calculated probabilities of their eligibility. Two reviewers independently screened the prioritized records presented by ASReview. Screening continued until 300 consecutive citations were judged as irrelevant, indicating that the probability of identifying relevant studies from remaining unscreened studies was extremely low. The use of ASReview ensured that the relevant studies are identified, while significantly reduced the workload associated with clearly irrelevant records.

Following title–abstract screening, the same two reviewers independently conducted full-text eligibility assessments of studies that were relevant according to the ASReview screening. Any disagreements between the two reviewers were resolved by discussion or the involvement of a third reviewer. Multiple reports of the same study were linked and treated as a single study unit. For data extraction, we prioritized the most recent or complete reports to avoid double-counting of participants in the meta-analysis.

### Data extraction and quality assessment

We used a standardized extraction form to obtain the following data from the selected studies: bibliographic details (authors, year of publication, country of study), study objectives, study design, study participants, experimental and control interventions, outcome measures, and main study findings. The quality of, and risk of bias in, included studies was assessed using the JBI Critical Appraisal Checklist for Randomized Controlled Trials [17], and JBI Checklist for Quasi-Experimental or non-Randomised studies [18]. Two reviewers independently conducted the data extraction and quality assessment, and disagreements were resolved through discussion or consultation with a third reviewer.

### Data synthesis and statistical analysis

Included studies reported different categories of outcomes (binary or continuous), and used different methods to measure effects of interventions. To combine results of different types of outcome measures, we used standardised mean difference (SMD) or *Cohen’s d*: [19] *Cohen’s d = (difference in means)/(pooled standard deviation).* Where necessary, we converted estimated odds ratio or correlation coefficients to SMD according to methods suggested by the Campbell Collaboration [20]. Empirical evidence indicates that these methods are methodologically acceptable and practically useful [21]. The SMD reflects effect size relative to the variability observed in studies, which may be difficult to interpret as its unit was not the original unit of measurement in studies. Cohen suggested that SMD = 0.2 to 0.5 represents a small effect, 0.5 to 0.8 a moderate effect, and > 0.8 a large effect [19]. In this meta-analysis, we applied random-effects model to quantitatively pooling data on the primary outcomes, effects of AI tools on documentation time and clinician burnout, using the R “meta” package [22]. Results of individual studies were weighted by the inverse of a combination of with-in study variance and between-study variance.

Heterogeneity in meta-analyses was statistically tested and measured using the *I*^2^ statistic. The interpretation of estimated *I*^2^ statistics was according to the Cochrane Collaboration’s rough guide: 0% to 40% may not be important; 30% to 60% may represent moderate heterogeneity; 50% to 90% may represent substantial heterogeneity; and 75% to 100% may represent considerable heterogeneity [23]. Asymmetry in funnel plots was statistically tested using Egger’s test, where a meta-analysis included at least 10 primary studies [24]. To explore the influence of study-level factors on the results, we conducted subgroup analyses by study design, type of tasks (consultation or other), whether AI-generated documents were edited by clinicians, and whether the evaluation was conducted in real clinical practice (real patient or simulated). Statistical significance was defined as *p* < 0.05 in this meta-analysis.

When a study provided data on multiple documentation burden-related outcomes, the outcome measure with the smallest effect size was chosen for the meta-analysis. From various time-related outcomes in the same study, the time spent on writing clinical notes was preferred. Due to substantial heterogeneity and inconsistency regarding secondary outcomes on utilization and accuracy of AI tools, we only provided a narrative synthesis of key findings.

## Results

**Fig. 1:**
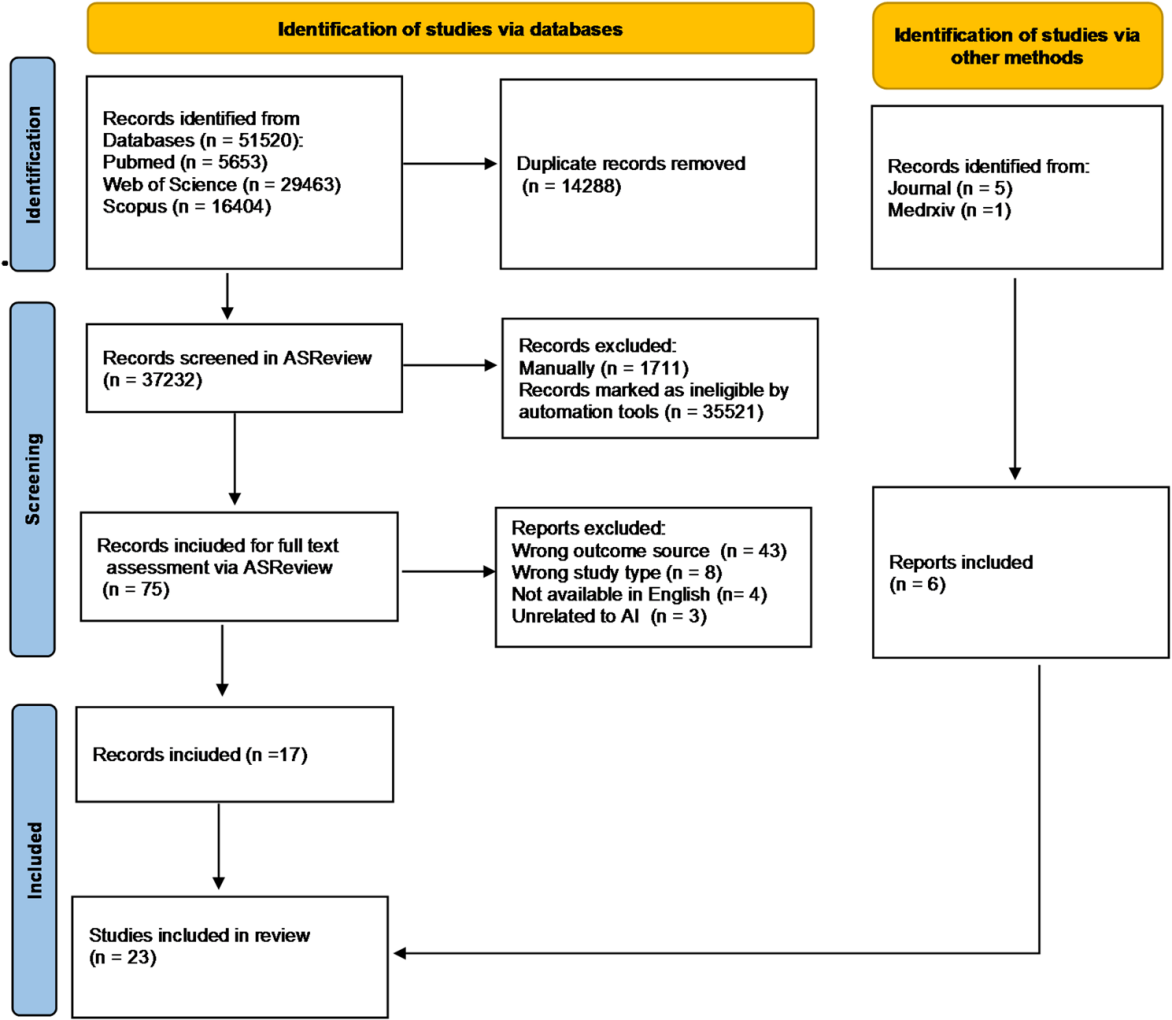
PRISMA flow diagram for a systematic review of effects of AI/LLM on clinical documentation burden

The PRISMA diagram for literature search and study selection is shown in Fig. 1. After removing duplicate records from searches of multiple bibliographic databases, we screened 37,232 references, and selected 75 studies for full text assessment, and initially included 17 studies. By manually checking references of retrieved articles and contents of several journals, 6 additional studies were identified. We eventually identified 23 relevant studies [7, 25–46]. 

The main characteristics of the included studies are summarized in Table 1 (further details on the extracted data can be found in Supplementary file-2), and results of quality assessment are provided in Supplementary file-3. Of the 23 studies included, 12 were non-randomised studies with a parallel control group [26, 27, 31–35, 40, 41, 44–46], while 11 employed a before-and-after comparison design [7, 25, 28–30, 36−39, 42, 43]. The main methodological issues identified were unclear comparability of participants (52.2%), the absence of current control groups (47.8%), incomplete follow-up (52.2%), lack of multiple measurements of the outcome both before and after the intervention (100%), and unclear reliability of the measured outcomes (65.2%). Most studies included were of rather small sample sizes (Table 1).

Of the 23 studies included, 18 (78.3%) were conducted in the United States, two in Germany, one was a joint study between Sweden and Switzerland, and one each was conducted in the Netherlands and the UK. ChatGPT-4.0 was employed for clinical documentation purposes in six studies, utilizing prompts crafted to follow the required structure and style (Table 1 and supplementary file − 2). Additionally, AI tools designed specifically for clinical documentation tasks were implemented in 17 studies, with 9 of these using Nuance Dragon Ambient eXperience (DAX) and 3 studies using Abridge systems. Speech-to-text (automatic speech recognition) technologies were involved in 19 studies of AI-generated clinical notes after patient-clinician encounters.

The study participants varied and included primary care clinicians, pediatricians, ICU specialists, among others, from a wide range of different medical settings (Table 1). The median number of study participants was 40, ranged greatly across studies from 4 resident physicians at a psychiatric clinic [33] to approximately 10,000 clinicians in an integrated health care delivery system [25, 33]. Regarding the nature of data types, 16 studies directly collected data from real clinical practices, while 7 utilized fictional or simulated cases. In terms of clinical tasks, 20 studies focused on clinical notes post-consultation, two on discharge summaries, and one on replies to patient inbox messages. AI-generated clinical notes were reviewed and edited by clinicians in 20 studies, while in the remaining three studies, which used fictional data, the notes were not explicitly reviewed or edited by clinicians (Table 1).

**Table 1:** The main characteristics of included studies on AI application in clinical documentation. BAC refers to before-and-after comparison; NRS, non-randomized study; AI, artificial intelligence; LLM, large Language model; NLP, natural Language processing; EHR, electronic health records. DAX, Dragon ambient eXperience. GPT, generative pretrained Transformers

Study & country	AI-tool used	Setting and participants	Design: sample size	Data, task, draft edited or not
Albrecht 2025 (USA)	Abridge: a smart-phone app to record patient-clinician conversations, and clinicians used a web editor to view and edit the AI-drafted note.	· Medical Center setting · Clinicians from 30 medical specialties including primary care, medical subspecialty, and surgical subspecialty	BAC: pre- (93/181) and post-(99/133) implementation survey.	· Real practice · Consultation · Edited: yes
Balloch 2024 (UK)	GPT-4.0; using prompt for a standardised note template and style.	· Hospital setting · Medical consultants and allied health professionals	NRS: 8 clinicians conducted 47 (including 24 AI-based) simulated consultations.	· Simulated · Consultation · Edited: yes
Barak-Corren 2024 (USA)	GPT-4.0; prompts included both structured and unstructured components.	· Pediatric emergency department · Attending physicians	NRS: 10 paediatric emergency medicine physicians with 40 supervisory notes.	· Simulated · Consultation · Edited: yes
Cao 2025 (USA)	Ambient scribe tool integrated with EHR (DAX Copilot; Nuance).	· Academic and community-based dermatology clinics · Dermatologists and physician assistants	BAC: 12 clinicians (10 dermatologists and 2 physician assistants).	· Real practice · Consultation · Edited: yes
Duggan 2024 (USA)	Ambient scribe tool integrated with EHR (DAX Copilot; Nuance).	· An academic health system · Physicians, nurse practitioners, and physician assistants, from outpatient departments	BAC: 46 clinicians with a 2-week training period followed by 5 weeks of ambient scribe usage.	· Real practice · Consultation · Edited: yes
Galloway 2024 (USA)	Abridge: Ambient listening and generative AI technology.	· An urban integrated academic medical institution · Specialists including primary care, advanced practice professionals, and other specialities	BAC: 31 clinicians who completed both the onboarding and follow-up (60 days after) web-based surveys.	· Real practice · Consultation · Edited: yes
Garcia 2024 (USA)	GPT-4 for drafting replies to patient inbox messages; prompt included the patient message, selected structured data elements and the last clinic note.	· An academic medical center · Attending physicians, advanced practice practitioners, clinic nurses, and clinical pharmacists from the divisions of primary care and gastroenterology and hepatology	BAC: 162 clinicians participated; 73 completed both the pre- & post-survey.	· Real practice · Reply to inbox message · Edited: yes
Haberle 2024 (USA)	DAX Copilot; specifically developed by vendor	· A large medical group · Participants were from adult and pediatric primary care, orthopedics, sports medicine, allergy, endocrinology, rheumatology, cardiology, neurology, neurosurgery, OB/GYN, oncology, urology, otolaryngology, and psychiatry	NRS: Of the 99 participants, 76 matched control group providers were included for analysis.	· Real practice · Consultation · Edited: yes
Hudson 2025 (USA)	Abridge - an electronic health record-integrated ambient AI platform.	· A large academic institution · Ambulatory providers	NRS: 40 ambulatory providers, each had 2 clinic sessions, and were randomized to first use either Abridge or usual note writing process.	· Real practice · Consultation · Edited: yes
Janota 2024 (Germany)	GPT-4.0; received a five-minute training with two sample discharge reports	· Psychiatric clinic · Resident physicians and psychotherapists	NRS: 8 reports, including 2 AI-generated, was assessed by 4 attending physicians.	· Simulated · Discharge summary · Edited: not or unclear
Kaufman 2016 (USA)	The Medical Language Extraction and Encoding System (MedLEE) accepts unstructured clinical text inputs and outputs structured clinical information in a variety of formats.	· University medical center · Neurologists, cardiologists, and nephrologists	NRS: 31 participants, and a total of 118 notes across the 3 subject areas.	· Simulated · Consultation · Edited: yes
Liu 2024 (USA)	DAX Copilot; specifically developed by vendor.	· Outpatient clinics within Atrium Health · Family medicine, internal medicine, and general pediatrics clinicians	NRS: 112 primary care clinicians using DAX and 103 without using DAX.	· Real practice · Consultation · Edited: yes
Ma 2024 (USA)	DAX Copilot; specifically developed by vendor	· An academic medical center · Physicians from ambulatory disciplines	BAC: 45 physicians from 8 ambulatory disciplines over 3 months.	· Real practice · Consultation · Edited: yes
Misurac 2024 (USA)	Nabla Copilot; specifically developed by vendor.	· University medical center · Ambulatory physicians and advanced practice providers	BAC: Pre- & post-surveys were completed by 35/38 participants.	· Real practice · Consultation · Edited: yes
Nguyen 2023 (USA)	Dragon Ambient eXperience (DAX) versions 3.0.5, 3.0.6, and 3.0.7 (Nuance: Burlington, MA)	· A National Cancer Institute (NCI) · Clinicians with various subspecialties, including endocrine, GI oncology, genetic counselling, sarcoma, and internal medicine	BAC: a survey at baseline and 1 month after using the DAX. 10/21 participants responded to the baseline survey, and 9/21 to the 1-month survey. 8 participants completed the interview.	· Real practice · Consultation · Edited: yes
Owens 2024 (USA)	DAX Copilot; specifically developed by vendor	· A community teaching health system · Primary care providers	BAC: 83 participants completed the survey; 19 provided data in the analysis of documentation time.	· Real practice · Consultation · Edited: yes
Peine 2023 (Germany)	Mona system; specifically developed by vendor	· Intensive care units (ICUs) · Medical professionals, including physicians, medical students after their fourth study year, and nurses	NRS: 60 ICU-experienced medical professionals.	· Simulated · Consultation · Edited: not or unclear
Rosenberg 2024 (Sweden & Switzerland)	GPT-4.0; Prompt formulated by 2 authors	· University hospitals · Orthopedic surgery residents	NRS: 6 fictional cases with 12 documents in total.	· Simulated · Discharge summary · Edited: not or unclear
Shah 2024 (USA)	DAX Copilot; specifically developed by vendor	· An academic medical center · Primary care and ambulatory specialties	BAC: Of the 48 physicians enrolled in the pilot, 38 were included in the paired pre- and post-survey analysis.	· Real practice · Consultation · Edited: yes
Stults 2025 (USA)	Ambient AI documentation platform (Abridge AI, Inc.)	· A large health care organization · Clinicians including primary care, medical and surgical subspecialties	BAC: Of 100 clinicians, 92 clinicians had EHR metrics, and 57 completed both pre- and post-implementation surveys.	· Real practice · Consultation · Edited: yes
Tierney 2025 (USA)	Ambient AI scribe; specifically developed by vendor	· A large multidisciplinary physician group · Physicians with a wide range of different specialties	NRS: Of the 10,000 physicians and staff, 7260 physicians enabled the tool in a total of 2,576,627 encounters across a wide array of medical specialties and locations.	· Real practice · Consultation · Edited: yes
van-Buchen 2024 (Netherlands)	GPT-4 (fine-tuned), GPT-3.5 Turbo; Prompt with tailored structure and additional rules for summarization	· University medical center · Medical students with experience in clinical practice and clinical documentation	NRS: 156 manual, 137 automatic, and 137 edited summaries from 21 medical students.	· Simulated; · Consultation; · Edited: yes
Zuchowski 2022 (Germany)	Indicda easySpeak (DFC Systems, Aschheim, Germany) and Dragon Naturally Speaking (Nuance Communications, Massachusetts, USA) software.	· Hospital setting · Clinicians from the nephrology, haematology and emergency departments	NRS: 15 clinicians participated over a period of 6 months, producing 163 samples used speech recognition software and the remaining 150 used typing.	· Real practice · Consultation · Edited: yes

### Effects of AI tools on documentation burden

**Fig. 2:**
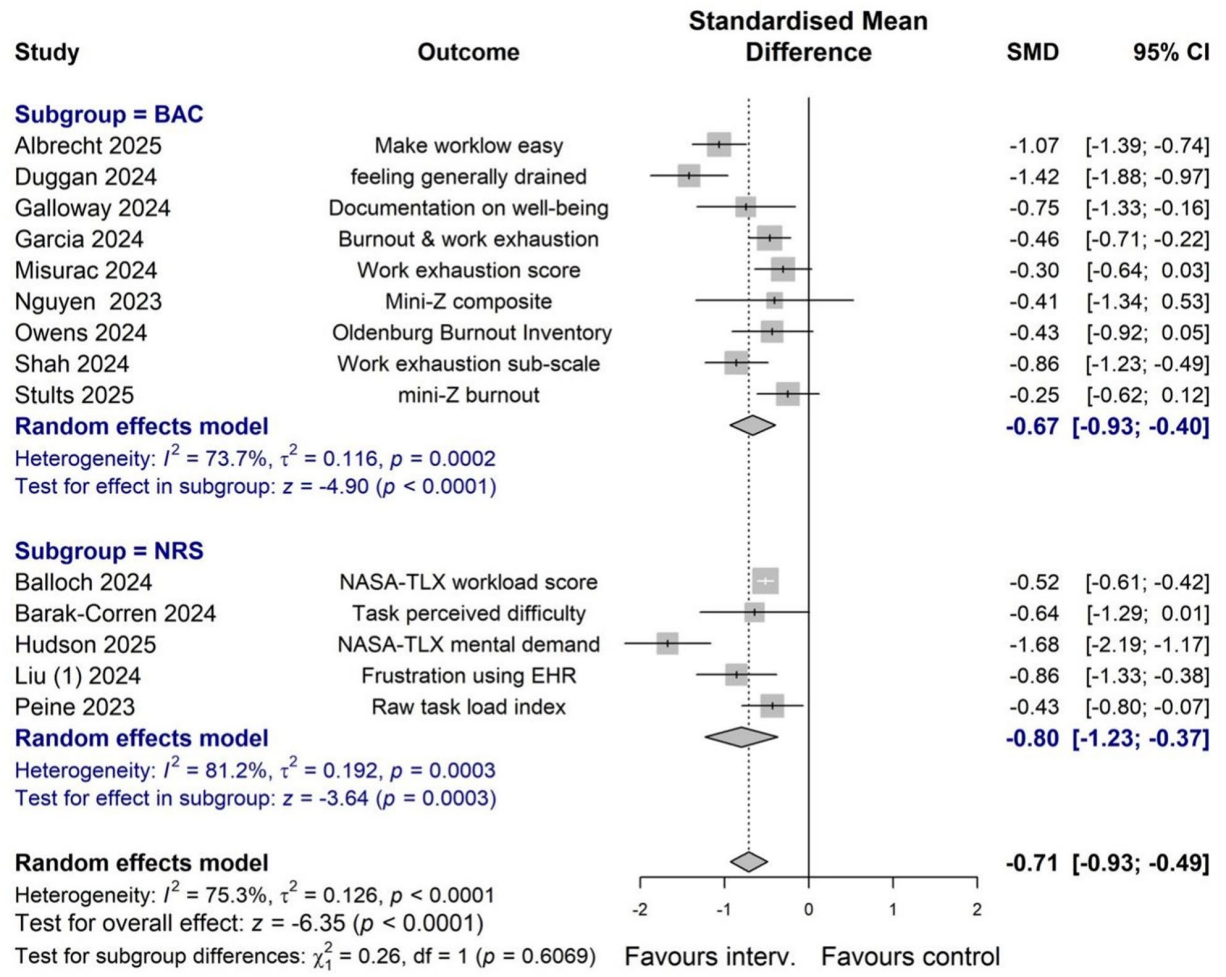
Forest plot of effect of AI vs. control on clinical documentation burden outcomes. Notes: BAC, before-after comparison. NRS, non-randomized control study

Effects of AI applications on documentation burden were evaluated in 15 studies, including 5 non-randomized studies (NRS) and 10 before-and-after comparison studies. Documentation burden were evaluated using the National Aeronautics and Space Administration Task Load Index (NASA-TLX) [7, 26, 40, 42], perceived task difficulty [27], frustration using EHR [35], the Stanford Professional Fulfilment Index (PFI) [38], or the Oldenburg Burnout Inventory (OLBI) [37]. Fig. 2 shows results of a meta-analysis with subgroup analysis by design. There was significant heterogeneity in results across studies (*I*^*2*^ = 75.3%, *p* < 0.001), although the difference between subgroups by design was statistically non-significant (*p* = *0.61*). The analysis pooling results of the 14 studies yielded an overall standardized mean difference (SMD) of -0.71 (95% confidence interval [CI]: -0.93 to -0.49). This suggests a moderate reduction in documentation workload and related burnout when using AI tools compared to control methods. The estimated SMD= -0.71 can be converted to an odds ratio (OR) of 0.28, or a risk ratio (RR) of 0.39 if assuming a baseline physician burnout prevalence of 40% [1].

Table 2 further details the meta-analysis results across different subgroups, categorized by application of ChatGPT or specially designed tools, by real patient care practice or fictional cases, by consultation or other tasks, and whether the AI-generated drafts were edited by clinicians. In general, AI tools alleviated documentation burden, regardless of the study design, data origin, task involvement, or the editing status of drafts. No significant differences were detected between subgroups by study design (*p* = 0.61), and by editing of AI-drafted notes or not (*p* = 0.17). Estimated effect sizes were larger for patient consultation tasks, compared with other clinical tasks (*p* = 0.06). Furthermore, effects by using general GPT tended to be smaller than specially designed AI tools (*p* = 0.08), and effects from studies in real practice tended to be greater than those based on fictional cases (*p* = 0.08).

Funnel plots for documentation burden and time outcomes are available in Supplementary file − 4. Funnel plot asymmetry for documentation burden outcomes was statistically non-significant (Egger’s test *p* = 0.17).

Subgroup analyses suggested that purpose-built ambient systems showed greater benefits than repurposed generative AI tools, and that rules-based AI produced more consistent outcomes than probabilistic generative models.

**Table 2:** Effects of AI application on Documentation burden outcomes by subgroups. BAC refers to before-and-after comparison. NRS, non-randomized study. Specially designed tools refer to AI-tools specially designed to be used in clinical practice by clinicians to create draft clinical notes. Consultation refers to the creation of clinical notes following a clinician-patient encounter. Other tasks refer to the creation of reply to inbox message and discharge summaries

Subgroup	No. of studies	SMD (95% CI)	Heterogeneity: I^2^; *p*-value	Test for subgroup differences
Total	14	-0.71 (-0.93; -0.49)	75.3%; *p* < 00001	-
By study design:				
NRS	5	-0.80 (-1.23; -0.37)	81.2%; *p* < 0.001	*p* = 0.61
BAC	9	-0.67 (-0.93; -0.40)	73.7%; *p* < 0.001	
By GPT or specially designed tools				
GPT	3	-0.51 (-0.60; -0.42)	0%; *p* = 0.86	*P* = 0.08
Specially designed	11	-0.77 (-1.05; -0.49)	77.3%; *p* < 0.0001	
By real-practice or not:				
Real practice	11	-0.77 (-1.05; -0.50)	78.2%; *p* < 0.0001	*p* = 0.08
Fictional/simulated	3	-0.51 (-0.60; -0.42)	0.0%; *p* = 0.84	
By consultation or other:				
Consultation	12	-0.77 (-1.02; -0.51)	78.4%; *p* < 0.0001	*p* = 0.06
Other tasks	2	-0.45 (-0.66; -0.25)	0.0%; *p* = 0.89	
By editing of AI draft or not:				
Edited	13	-0.74 (-0.97; -0.50)	76.9%; *p* < 0.0001	*p* = 0.17
Not/unclear	1	-0.43 (-0.80; -0.07)	-	

### Effects of AI tools on documentation time

**Fig. 3:**
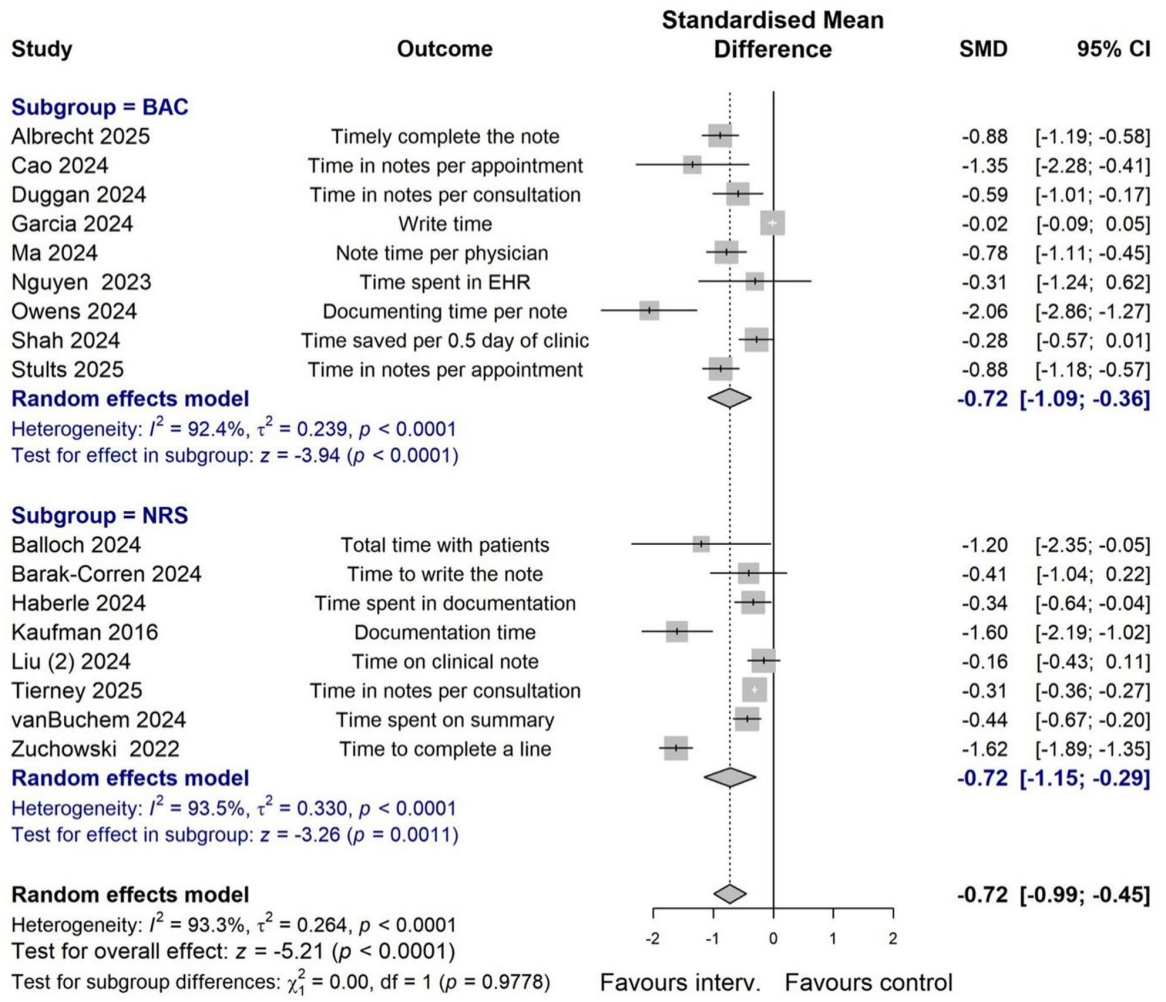
Forest plot of effect of generative AI vs. control on clinical documentation time outcomes. Excluding studies without editing of AI-generated draft notes. SMD refers to standardized mean difference. BAC refers to before-and-after comparison. NRS refers to non-randomized study

Given that clinicians should review and edit AI-generated notes in actual healthcare practice, our meta-analysis on documentation time outcomes focused on 17 studies that included this editing process (Table 3; Fig. 3). Different methods were used to measure changes in documentation time outcomes, including time spent on preparing clinical notes, documentation after normal work hours or outside scheduled hours, and patient consultation time. When multiple time outcomes were present in a study, the time taken to create clinical notes (including review and editing) was adopted in the meta-analysis. Results shows that there was considerable heterogeneity across studies (*I*^*2*^ = *93.3%*; *p* < 0.0001), and the use of AI tools significantly reduced documentation time (-0.72, 95% CI -0.99 to -0.45). We found no significant differences between subgroups by study design (test for subgroup difference *p* = 0.98), or by real patient practice vs. fictional cases (*p* = 0.63). However, the use of specially designed AI tools showed a relatively larger effect on time savings compared to general GPT technologies (*p* = 0.04). Although 16 studies indicated a notable decrease in documentation time after consultations, one study [7] reported no significant change in the time spent responding to patient inbox messages.

Funnel plot asymmetry for time saving outcomes is statistically significant (Egger’s test *p* = 0.02; Supplementary file − 4).

**Table 3:** Effects of AI application on Documentation time by subgroups, after excluding studies without editing of AI-generated draft notes. BAC refers to before-and-after comparison. NRS, non-randomized study. Specially designed tools refer to AI-tools specially designed to be used in clinical practice by clinicians to create draft clinical notes. Consultation refers to the creation of clinical notes following a clinician-patient encounter. Other tasks refer to the creation of reply to inbox message and discharge summaries

Subgroup	No. of studies	SMD (95% CI)	Heterogeneity: I^2^; *p*-value	Test for subgroup differences
Total	17	-0.72 (-0.99; -0.45)	93.3%; *p* < 0.0001	-
By study design (studies with AI draft edited)				
NRS	8	-0.72 (-1.15; -0.29)	93.5%; *p* < 0.0001	*p* = 0.98
BAC	9	-0.72 (-1.09; -0.36)	92.4%; *p* < 0.0001	
By using GPT or specially designed tools (studies with AI draft edited)				
GPT	4	-0.32 (-0.66; 0.03)	81.8%; *p* < 0.0001	*p* = 0.04
Specially designed	13	-0.81 (-1.13; -0.50)	92.4%; *p* < 0.0001	
By real-patient data or not (studies with AI draft edited)				
Real-patient data	13	-0.69 (-1.00; -0.38)	94.4%; *p* < 0.0001	*p* = 0.63
Fictional/simulated	4	-0.86 (-1.47; -0.24)	79.4%; *p* = 0.002	
By consultation or other (studies with AI draft edited)				
Consultation	16	-0.77 (-1.04; -0.50)	90.6%; *p* < 0.0001	*p* < 0.0001
Other task	1	-0.02 (-0.09; 0.05)	-	

### Effects of AI applications on documentation quality

The quality of AI-generated clinical notes was evaluated across 10 studies, including one using the Sheffiled Assessment Instrument for Letters (SAIL) [26], three employing a modified Physician Documentation Quality Instrument (PDQI-9) [6, 34, 45], and others utilizing informal quality assessment methods [27, 33, 38, 40, 41, 46]. The findings from these studies consistently demonstrated that the quality of AI-generated clinical documents was at least comparable to those written by clinicians (Appendix-4). However, the results of these studies also revealed that AI-generated clinical documents may contain inaccuracies and errors; thus, clinicians’ review and editing are necessary in real-world healthcare practices.

## Discussion

The primary studies collated in this systematic review exhibited considerable heterogeneity concerning their design, settings, clinical specialties, and outcome measures employed. The results revealed a moderate impact of AI tools on reducing the workload and burnout among healthcare professionals. The application of AI tools can reduce the odds of burnout related to clinical documentation by 72% (OR = 0.28). In addition, AI applications in clinical documentation resulted in considerable time savings.

Findings from this meta-analysis are qualitatively consistent with those from some narrative reviews, indicating that AI applications resulted in reduced workload and burnout [11–14]. The reduction in documentation burden by AI applications may be attributed to their ability to ease cognitive workload, even without shortening total time required [47]. Clinicians, without the assistance of AI tools, are required to juggle multiple mental tasks simultaneously, such as listening to patient complaints, writing down notes during conversations, and later drafting and formalizing structured clinical notes from scratch post-consultation. When combined with speech recognition technology, natural language processing and large language models, AI tools can instantly generate a structured preliminary note right after patient-physician conversations. Although the draft notes still necessitate review and editing by clinicians, the significant reduction in cognitive effort contributes to lessened documentation burden and burnout.

The included studies were of low methodological quality, including lack of randomized controlled trials (RCTs), small sample sizes, and variation in outcome measures. In addition, publication bias could not be ruled out, as funnel plot for time saving outcomes was statistically significantly asymmetric. Even so, it may be reassuring that studies with a concurrent control or using data from real practices tended to report similar or larger effects compared with those without a concurrent control or using fictional cases. Implementation of AI tools for clinical documentation in real healthcare practice is a complex organizational intervention, involving multiple process components. High-quality RCTs are possible but less likely used to evaluate organisational complex interventions, so that policy makers may often have to rely on evidence from observational or natural experimental studies. Making the issue more complex is that AI tools for clinical documentation are continuously evolving, along with the rapid advancements in AI and LLM technologies. Therefore, further studies of AI applications for clinical documentation may be conducted according to the dynamic nature of LLMs, and “making possible complex medical AI systems which continuously learn and adapt in situ from new data and interactions with users while enabling continuous real-time monitoring and clinical validation” [48].

Our systematic review focused on the impact of AI applications on documentation burden, rather than a comprehensive assessment of the quality of AI-generated clinical notes. The evidence from the studies included in our systematic review indicated that the quality of notes generated by AI tools was at least comparable to those prepared manually by clinicians, consistent with findings from other systematic reviews [11–14]. Although AI-generated notes often meet the quality of those created manually, it is crucial to recognize that errors, inaccuracies and unsuitable contents still remain. A recently published study found that LLM-generated discharge summary narratives were “more likely to contain errors but had low overall harmfulness scores” [49]. In practical settings, it is essential for clinicians to review and edit AI-generated clinical documents before finalization. Furthermore, the effectiveness of AI tools is intrinsically linked to the maturity and quality of the existing digital documentation systems. The transition from analog (paper-based) to electronic medical records (EMR) has previously demonstrated that digitization alone does not guarantee reduced burden; rather, its impact depends heavily on the implementation process, system interoperability, and user adoption [50, 51]. Wurster et al. highlighted that while EMRs can improve data completeness, poorly integrated systems (e.g., data silos) may lead to double documentation and fragmented workflows [51]. Consequently, AI cannot be viewed as a standalone solution but requires a well-implemented digital infrastructure. Only robust, interoperable digital systems offer sufficient conditions for the successful integration of AI tools to genuinely alleviate clinical documentation burden rather than adding technical complexity.

Our meta-analysis has other limitations. We only included studies published in the English language, which means we might have overlooked research available in other languages. Secondly, our systematic review focused on AI applications for clinical note creation, although AI tools may also simultaneously assist EHR review, diagnostic and treatment recommendations, the coding and billing process [52]. Thirdly, our meta-analysis included studies that applied different AI technologies, including ambient vs. prompt-based systems, EHR-integrated vs. stand-alone tools, and rules-based vs. generative AI. However, the overall results consistently indicated that the application of AI technologies could reduce documentation related burden and time. Fourthly, the included studies were conducted mainly in the USA and other high-income countries, without data from low- and middle-income countries. Lastly, the AI tools evaluated in these studies might have been outdated due to the rapid advancements in AI technologies.

The implications of these findings are significant for the integration of AI and LLM tools in healthcare settings. They highlight the potential for these technologies to alleviate documentation burdens and, consequently, reduce the overall workload and burnout of healthcare professionals. However, the variability in results, presence of errors in AI-generated documents, and rapid advancement of AI technologies underscore the importance of continuous evaluation and improvement of these tools to ensure their efficacy and reliability in real-world applications [48]. 

In conclusion, AI technologies offer promising benefits for reducing clinical documentation burden. However, their implementation must be accompanied by rigorous quality control and ongoing evaluation in practical settings to optimize their effectiveness and safeguard patient care outcomes.

## Supplementary information

Below is the link to the electronic supplementary material.

Supplementary Material 1

Supplementary Material 2

Supplementary Material 3

Supplementary Material 4

## Data Availability

All data generated or analysed during this study are included in this article and its supplementary information files.

## Abbreviations

ACI: Ambient Clinical Intelligence
AI: Artificial Intelligence
ASReview: Automatic Systematic Reviews
BAC: Before-and-after comparison
CI: Confidence Interval
DAX: Dragon Ambient eXperience
EHR: Electronic Health Records
EMR: Electronic Medical Records
GPT: Generative Pretrained Transformers
JBI: Joanna Briggs Institute
LLM: Large Language Model
ML: Machine Learning
NLP: Natural Language Processing
NRS: Non-randomized study
OR: Odds Ratio
PRISMA: Preferred Reporting Items for Systematic Reviews and meta-analyses
PROSPERO: International prospective register of systematic reviews
RCT: Randomized Controlled Trial
RR: Risk Ratio
SMD: Standardized Mean Difference
