# Supplementary material for: Application of artificial intelligence tools and clinical documentation burden: a systematic review and meta-analysis
Source: BMC Med Inform Decis Mak. 2025 Dec 24;26:29. doi: 10.1186/s12911-025-03324-w (PMC12836966; doi:10.1186/s12911-025-03324-w)
Supplement: Supplementary file 1 — Supplementary Material 1 [file 12911_2025_3324_MOESM1_ESM.pdf]

Supplementary file -4

Funnel plots for meta-analyses of documentation burden and time outcomes

A. Funnel plots for documentation burden outcomes

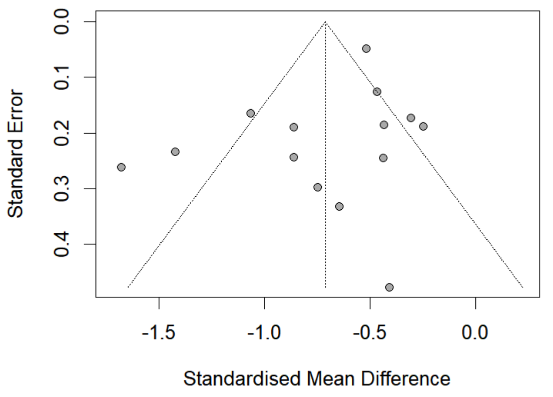

Linear regression test of funnel plot asymmetry

Test result: t = -1.48, df = 12, p-value = 0.1658
Bias estimate: -1.2652 (SE = 0.8573)

Details:
- multiplicative residual heterogeneity variance (tau^2 = 3.7161)
- predictor: standard error
- weight: inverse variance
- reference: Egger et al. (1997), BMJ

B. Funnel plots for documentation burden outcomes

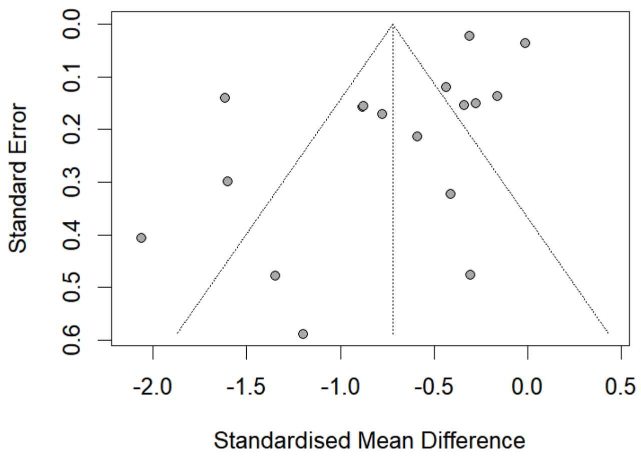

Linear regression test of funnel plot asymmetry

Test result: t = -2.63, df = 15, p-value = 0.0191
Bias estimate: -2.7011 (SE = 1.0288)

Details:
- multiplicative residual heterogeneity variance (tau^2 = 10.8589)
- predictor: standard error
- weight: inverse variance
- reference: Egger et al. (1997), BMJ
