# Supplementary material for: Application of artificial intelligence tools and clinical documentation burden: a systematic review and meta-analysis
Source: BMC Med Inform Decis Mak. 2025 Dec 24;26:29. doi: 10.1186/s12911-025-03324-w (PMC12836966; doi:10.1186/s12911-025-03324-w)
Supplement: Supplementary file 2 — Supplementary Material 2 [file 12911_2025_3324_MOESM2_ESM.pdf]

Supplementary file -3:

Quality assessment of included studies (non-randomised studies and before-after comparison studies)

**Author (yr)**	Q1	Q2	Q3	Q4	Q5	Q6	Q7	Q8	Q9	Other limitations and notes
Albrecht 2025	Yes	Unclear	Unclear	No	No	No	No	Unclear	Yes	The pre- and post-implementation surveys differed in the number and content of questions, based on subjective perceptions of clinicians. Only 2 of the questions for each of the surveys were concordant enough to be compared with statistical analysis. The early adopter cohort of the AI ambient tool may have differed in clinical workload, comfort with technology, burnout, and documentation burden compared to non-participating clinicians.
Balloch 2024	Yes	Yes	Yes	Yes	No	Unclear	Yes	Unclear	Yes	Lack of a diverse and generalisable dataset for the cohort studied. Whilst the patient scenarios were realistic and spanned multiple clinical specialties, they may not represent highly complex consultations with large pre-existing volumes of medical record information.
Barak-Corren 2024	Yes	Yes	Unclear	Yes	No	Unclear	Yes	Unclear	Yes	Small sample size for proof-of-concept; the limited range of clinical scenarios; only considered the first output from GPT for each scenario.
Cao 2024	Yes	Unclear	Unclear	No	No	No	Unclear	Unclear	Yes	Limitations include the small size and single institution.
Duggan 2025	Yes	Yes	Unclear	No	No	Unclear	Yes	Yes	Yes	Bias introduced by participants' interest in the study may mean they are not representative of the wider clinician population. Future research would benefit from observing a larger sample size of physicians over a longer study period using a stable version of ambient scribing. There were weeks when clinicians performed outpatient charting in the EHR system but did not use ambient scribing for unknown reasons.
Galloway 2024	Yes	Yes	Unclear	No	No	No	Unclear	Unclear	Unclear	Limitations to this study include small sample sizes and a questionnaire change between the pre- and post-interven tion surveys, which may have impacted how some questions were interpreted.

Garcia 2024	Yes	Yes	Yes	No	No	Yes	Yes	Unclear	Yes	This was a single-group prospective study at a single institution, which limits the generalizability of results; limited to 2 departments and 4 types of clinicians. Regarding cognitive burden and burnout outcomes, novelty bias and the Hawthorne effect may have skewed toward positive results.
Haberle 2024	Yes	Yes	Unclear	Yes	No	Yes	Yes	Unclear	Yes	“Intention to treat” approach may be dampening the positive effects seen in outcomes. During implementation, factors such as note turnaround time, provider attrition, and scaling adoption could have introduced bias in these results. Survey data were unavailable for our control group.
Hudson 2025	Yes	Yes	Unclear	Yes	No	Yes	Yes	Yes	Unclear	A cross-over design
Janota 2024	Yes	Unclear	Yes	Yes	No	Yes	Yes	Unclear	Unclear	Small sample size (6 clinicians, 2 cases); fictional cases may lack real-world complexity. Minimal AI training (5 minutes). Potential bias: evaluators might recognize AI’s formal style.
Kaufman 2016	Yes	Unclear	Yes	Yes	No	Unclear	Yes	Yes	Yes	Physicians generated documentation for the study based on test scripts about fictitious patient encounters. The sample size for cardiology and nephrology was rather small owing to recruiting challenges. The results for time required to complete documentation may be biased toward free text and therefore in faviour of NLP Entry.
Liu (1,2) 2024	Yes	Unclear	Unclear	Yes	No	Yes	Yes	Unclear	Unclear	while we adjusted for a variety of factors, there will inherently be unmeasured confounding. We were not able to obtain comprehensive editing time in DAX from all participating clinicians because the vendor only had data starting midway through the study (July). A learning curve among DAX users that may have resulted in inefficiencies.
Ma 2024	Yes	Yes	Unclear	No	No	Unclear	Yes	Unclear	Yes	Limitations of this study include small sample size, volunteer and selection bias, potential impact from secular trends, inability to analyze time outcomes at the level of individual patient encounters, lack of comparison to alternate strategies, limitations to English-speaking patients only, and predominance of primary care physicians in our cohort.

Misurac 2024	Yes	Unclear	Yes	No	No	Yes	Yes	Yes	Yes	This was a single-center, observational, pre-post pilot study over a short period of time. Providers volunteered for the study and may not be representative of all providers.
Nguyen 2023	Yes	Unclear	Yes	No	No	No	Yes	Unclear	Unclear	(1) a single NCI-designated Compre hensive Cancer Center, precluding generalizations to other care settings. (2) A small number of participants, not statistically powered to detect differences in clinician well-being outcomes. (3) our study examined a subjective measure of documentation burden. Other clinician-level measures, such as frequency of DS use, timely note signoffs, and time spent documenting in EHR will be needed in future studies. (4) less than half of our pilot participants completed the surveys or interviews, which may imply ascertainment bias. (5) mainly included English-speaking patients.
Owens 2024	Yes	Unclear	Unclear	No	No	Unclear	Yes	Yes	Yes	A cross-sectional study unable to evaluate changes over time in burnout scores or compared to a control group. There might have been unidentified confounders between high and low users that may be associated with burnout. Time outcome was highly biased due to it was based on 19 participants with high implementation of DAX tool.
Peine 2023	Yes	Yes	Yes	Yes	No	Unclear	Yes	Unclear	Yes	The study was not performed in a real ICU setting due to infection constraints during the COVID-19 pandemic. The VIDS used in the study was in a prototypic stage. The tasks observed were predefined and may not fully represent the complexity of real-world ICU work. Implementing complex IT systems in healthcare workflows can produce logistical and economic challenges.

Rosenberg 2024	Yes	Unclear	Unclear	Yes	No	Yes	Yes	Unclear	Unclear	Only 6 fictional orthopedic cases; without a detailed human review of discharge documentations; the discharge notes were generated in English, which is not the resident’s native language. The use of fictional cases, while designed to reflect real-world scenarios, may not fully capture the complexities and nuances of actual patient data. While the expert panel was blinded, the physicians generating the documents were not, potentially introducing bias. Furthermore, the study only assessed the generation of discharge summaries and letters, not the time required for physician review and approval of the AI-generated documents, which is a crucial step for clinical implementation. Finally, the study did not explore the potential for bias in the training data of the LLM, which could influence the content generated.
Shah 2024	Yes	Unclear	Yes	No	No	Yes	Yes	Yes	Yes	The modest sample size as there were only 50 ambient AI scribe licenses available for this pilot. Physicians who expressed interest in using the tool were included, which may have introduced selection bias. The follow-up data were not comprehensively analyzed, which may affect the reliability of the long-term impact assessment. The study’s generalizability is limited due to the specific healthcare settings and participant demographics. Potential confounding variables, such as varying levels of technology acceptance among participants, were not fully controlled or explored.
Stults 2025	Yes	Yes	Unclear	No	No	Yes	Yes	Yes	Yes	These findings are from a single organization and so may not be generalizable. While preintervention and postintervention surveys were offered multiple times, there may have been response bias.

Tierney (2024, 2025)	Yes	Unclear	Unclear	Yes	No	Unclear	Yes	Unclear	Unclear	The study has several limitations: non-random, self-selected adoption of AI scribes, leading to selection bias; vendor changes that limited direct comparisons; unaccounted confounders like workflow and patient complexity; small survey sample sizes (102 physicians, 118 patients) affecting generalizability; limited data on note-editing and AI accuracy; and no assessment of equity impacts across patient groups, such as non-English speakers or those with disabilities.
van-Buchen 2024	Yes	Yes	Yes	Yes	No	Yes	Unclear	Unclear	Unclear	The study used mock consultations rather than real clinical setting, and the study setup was not fully representative of clinical practice. The students did not have a time cap for creating the summaries, while in clinical practice, physicians often have to create a summary during or in between consultations.
Zuchowski 2022	Yes	Unclear	Yes	Yes	No	Yes	Yes	Yes	Yes	Participation in this study was voluntary and participating clinicians were able to choose their method of note taking. This may have introduced a selection bias, with clinicians choosing the method they were already positively disposed to. Furthermore, the results may have been affected by factors that were not observable, such as individual variations in clinicians' note-taking ability and experience.

Assessment questions:

Q1 1. Is it clear in the study what is the 'cause' and what is the 'effect' (i.e. there is no confusion about which variable comes first)?
Q2 2. Were the participants included in any comparisons similar?
Q3 3. Were the participants included in any comparisons receiving similar treatment/care, other than the exposure or intervention of interest?
Q4 4. Was there a control group?
Q5 5. Were there multiple measurements of the outcome both pre and post the intervention/exposure?
Q6 6. Was follow up complete and if not, were differences between groups in terms of their follow up adequately described and analyzed?
Q7 7. Were the outcomes of participants included in any comparisons measured in the same way?
Q8 8. Were outcomes measured in a reliable way?
Q9 9. Was appropriate statistical analysis used?
