# Supplementary material for: Application of artificial intelligence tools and clinical documentation burden: a systematic review and meta-analysis
Source: BMC Med Inform Decis Mak. 2025 Dec 24;26:29. doi: 10.1186/s12911-025-03324-w (PMC12836966; doi:10.1186/s12911-025-03324-w)
Supplement: Supplementary file 3 — Supplementary Material 3 [file 12911_2025_3324_MOESM3_ESM.pdf]

Supplementary file -1: PubMed search strategy

#1 artificial intelligence [mh] OR “artificial intelligence”[tiab] OR "large language model*"[tiab] OR "machine learning"[tiab] OR "natural language processing"[tiab] OR "generative AI"[tiab] OR digital[tiab] OR automat*[tiab]

#2 burnout[tiab] OR burden[tiab] OR exhaustion[tiab] OR workload[tiab] OR "task load"[tiab] OR overload[tiab] OR “workplace stress”[tiab] OR “occupational stress”[tiab] OR “mental fatigue”[tiab]

3 documentation OR summarization OR "clinical documents"[tiab] OR "medical documents"[tiab] OR “clinical records”[tiab] OR "medical records"[tiab] OR "health records”[tiab] OR “patient records"[tiab] OR “clinical notes”[tiab] OR “medical notes”[tiab] OR “physician notes”[tiab] OR “nursing records”[tiab] OR “discharge summaries”[tiab] OR “discharge instructions”[tiab] OR “responses to patients”[tiab:~3] OR “letters to patients”[tiab:~3] OR scribe[tiab] OR Transcript*[tiab]

#1 AND #2 AND #3
