# Supplementary material for: Application of artificial intelligence tools and clinical documentation burden: a systematic review and meta-analysis
Source: BMC Med Inform Decis Mak. 2025 Dec 24;26:29. doi: 10.1186/s12911-025-03324-w (PMC12836966; doi:10.1186/s12911-025-03324-w)
Supplement: Supplementary file 4 — Supplementary Material 4 [file 12911_2025_3324_MOESM4_ESM.pdf]

Supplementary file -2:
Detailed data extracted from included studies

Author (yr)	Country	Objectives	Duration	Setting & healthcare workers	Document & patients	AI-technology & application
Albrecht 2025	USA	To evaluates the impact of an ambient artificial intelligence (AI) documentation platform on clinicians' perceptions of documentation workflow.	04/2023 - 03/2024	The University of Kansas Medical Center (KUMC), with clinicians from 30 medical specialties including primary care, medical subspecialty, and surgical subspecialty.	Preliminary draft clinical note for EHR following a clinician-patient encounter.	Abridge is an ambient AI platform that summarizes medical conversations for clinicians and patients across multiple care settings. Clinicians used an Abridge smartphone application to record patient-clinician conversations. Clinicians then use a web editor to view and edit the AI-drafted note. Once viewing and editing is complete, dot phrases are used to pull the draft note directly into the clini cian's note template in the EHR.
Balloch 2024	UK	To assess the clinical utility of an ambient AI tool in enhancing consultation experience and the completion of clinical documentation.	Not reported	Great Ormond Street Hospital (GOSH) in a simulated clinic environment, mimicking real-world outpatient consultations. Five medical consultants and three allied health professionals.	Each consultation required two completed documents, one clinic letter and one clinic note, within the allocated 20-min appointment. Outpatient consultations were simulated with professional medical actors playing the role of patients and parents.	AI tool to ambiently listen and summarise real-world clinical consultation audio into a clinic note and letter, using a pipeline of AI models including speech-to-text and LLMs (GPT-4 32K), guided by a 'prompt' (a standardised note template and style guidance) . Clinicians reviewed the documentation and made edits before transferring to the EHR.
Barak-Corren 2024	USA	To evaluate 3 uses for generative AI for clinical documentation in pediatric emergency medicine, measuring time savings, effort reduction, and physician attitudes and identifying potential risks and barriers.	Not reported	10 pediatric emergency medicine attending physicians from a single pediatric emergency department.	The 3 summaries included: a supervisory attending note, a structured handoff format, and a "letter to the family". 4 clinical scenarios were designed to reflect the breadth of care in pediatric emergency medicine, including 2 straightforward cases with a short past medical history, a few ancillary tests, and a narrow differential diagnosis and 2 intricate cases.	ChatGPT Version 4.0: Participants were asked to write a supervisory note for 4 clinical scenarios, 2 without any assistance and 2 with the assistance of ChatGPT. ChatGPT Plus (GPT-4 release May 3, 2023) was used to generate 3 distinct summaries from the resident's notes for each clinical scenario. Two pediatricians initially formulated prompts, with refinements made after a pilot interview with one pediatric emergency medicine attending physician. Each prompt used a blend of structured and unstructured components.
Cao 2024	USA	Pilot study assessing the impact on dermatologist workflow and patient encounters.	02/2021 - 01/2023	Academic and community-based dermatology clinics; dermatologists and physician assistants	Clinical consultation notes; dermatology patients	Dragon Ambient eXperience (DAX) (Nuance & Microsoft) as a digital scribe in . The application was selected due to easy integration with the EMR and ability to convert encounters into specialty-specific notes. DAX is AI driven, meaning that the software will learn to adjust notes based on clinician habits (language, formatting, etc) after a virtual scribe has dictated notes.

Author (yr)	Country	Objectives	Duration	Setting & healthcare workers	Document & patients	AI-technology & application
Duggan 2024	USA	To investigate the association of ambient scribing technology with efficiency, quality, and perceived burden of clinical documentation in the outpatient setting.	04/2024 - 08/2024	The outpatient setting of an academic health system in Philadelphia, Pennsylvania. Participants included physicians, nurse practitioners, and physician assistants, involving 17 different medical specialties	Preliminary draft clinical note for EHR following a clinician-patient encounter.	Clinicians used a commercially available ambient scribe tool (DAX Copilot; Nuance), integrated directly with the EHR. Clinicians were able to activate a recording button within the EHR mobile apppatient-visit interface to begin recording, and to stopp recording at the end of the visit. Afterwards, AI automatically interpreted the conversation and produced a full template or partial note section (eg, history of present illness, physical examination, assessment, and plan) to be available to the clinician to review most often in less than 1 minute.
Galloway 2024	USA	To evaluate the impact of Ambient listening technology on clinicians' documentation experience in the EHR and on overall well-being.	Not reported	Emory Healthcare, a large, urban integrated academic medical institution; clinicians included various specialists (primary care, advanced practice professionals, and other specialities)	Clinicians' documentation experience in the EHR	Abridge: Ambient listening technology is a tool that uses generative AI to generate a clinical note from spoken conversation between clinicians and patients during a scheduled encounter and is available for integration into an EHR's clinical workflows.
Garcia 2024	USA	To evaluate the implementation of a large language model used to draft responses to patient messages in the electronic inbox.	07/2023 - 08/2023	A single academic medical center (Stanford Health Care). Attending physicians, advanced practice practitioners, clinic nurses, and clinical pharmacists from the Divisions of Primary Care and Gastroenterology and Hepatology	Replies to patient portal messages	Stanford Medicine routed select patient messages upon arrival to the inbox messaging pool to EHR developer Epic for categorization via GPT-3.5 Turbo and GPT-4 for draft reply generation. Draft replies to patient portal messages were generated by a Health Insurance Portability and Accountability Act– compliant EHR– integrated LLM. Patient messages and draft replies were displayed within the HER with options to start withdraft or start blank reply
Haberle 2024	USA	To assess the impact of the use of an ambient listening/digital scribing solution (Nuance Dragon Ambient eXperience (DAX)) on care giver engagement, time spent on Electronic Health Record (EHR)	03/2022 - 09/2022	A subset of clinics in the Intermountain Medical Group in Utah. Participants were randomly selected from adult and pediatric primary care, orthopedics, sports medicine, allergy, endocrinology, rheumatology, cardiology, neurology, neurosurgery, OB/GYN, oncology, urology, otolaryngology, and psychiatry.	Preliminary clinical note for editing and parsing to EHR.	Nuance Dragon Ambient eXperience (DAX) leverages ambient listening, conversational AI, and generative AI technology to create outpatient provider clinical documentation. From the output of the conversational AI, a generative AI-derived draft note is made available to Nuance and their human quality reviewers.
Hudson 2025	USA	This study evaluated the effect of an ambient AI documentation platform on clinician cognitive load.	03/2024 - 04/2024	Ambulatory providers at a large academic institution.	Preliminary clinical notes for EHR following clinician-patient encounters	Abridge - an electronic health record-integrated ambient AI platform

Author (yr)	Country	Objectives	Duration	Setting & healthcare workers	Document & patients	AI-technology & application
Janota 2024	Germany	To evaluate the quality of discharge summaries produced by clinical staff and by an AI model (ChatGPT 4.0).	Not reported	A psychiatric clinic. Resident physicians (n=3) and psychotherapists (n=3).	Discharge summaries. Two fictional patients were created using standard admission forms and supplemented with fictional disease progressions. Case 1 involved a patient with depressive disorders, sleeping pill dependency, and hypertension, totaling 577 words; Case 2 was a patient with paranoid schizophrenia, alcohol dependency, and arterial hypertension, totaling 394 words.	Data on the two fictional patients were given to ChatGPT 4.0 to create a standardized discharge report. ChatGPT 4.0 received a five-minute training with two sample discharge reports.
Kaufman 2016	USA	To evaluate an NLP-enabled data capture method using dictation and data extraction from transcribed documents (NLP Entry) in terms of documentation time, documentation quality, and usability versus standard EHR keyboard-and-mouse data entry.	Not reported	The Columbia University Medical Center's (CUMC), physician participants were recruited through referrals. The 31 participants included neurologists, cardiologists, and nephrologists.	The test scripts were based on anonymized transcription documents that were modified by 4 expert clinicians (2 fellows and 2 attending physicians). After reviewing test scripts that described cases of the fictitious patients, the participants generated 4 multisectional consultation or admission notes using 4 documentation protocols.	The Medical Language Extraction and Encoding System (MedLEE) accepts unstructured clinical text inputs and outputs structured clinical information in a variety of formats. It enables a workflow by which (1) the physician dictates, (2) the dictation is transcribed or subjected to speech recognition, (3) MediSapien NLP generates structured data from the transcription, and (4) the structured data and text are inserted into the EHR.
Liu (1, 2) 2024	USA	(1) To evaluate clinicians' experiences with clinical documentation before and after implementing an AI-powered clinical documentation tool. (2) To determine whether using DAX improves efficiency for clinicians (as measured by EHR use metrics) and financial performance for the health system.	06/2023 - 08/2023	All outpatient clinics in North Carolina and Georgia within Atrium Health; including family medicine, internal medicine, and general pediatrics clinicians (physicians and advanced practice practitioners).	Preliminary clinical note for EHR following a clinician-patient encounter.	Nuance's Dragon Ambient eXperience (DAX) Copilot is an electronic health record (EHR)–integrated AI-enabled scribe software. It synthesizes a preliminary outpatient clinical note by "listening" to the conversation between a clinician and a patient during their visit. Participants used the PowerMic Mobile DMO/DAX application on smartphones to capture the dialogue. Once the visit is complete, the clinician turns off the recording and DAX drafts the note within 30 seconds. Clinicians transfer the note to the EHR through voice command or the "Copy" (partial or all) button and edit within the EHR before accepting the note.

Author (yr)	Country	Objectives	Duration	Setting & healthcare workers	Document & patients	AI-technology & application
Ma 2024	USA	To quantify utilization and impact on documentation time of a large language model-powered ambient artificial intelligence (AI) scribe.	10/2023 - 01/2024	Ambulatory settings at a single academic medical center (Stanford Health Care).	Preliminary clinical notes for EHR following clinician-patient encounters, including all ambulatory encounters (including procedural and virtual encounters) performed by pilot physicians.	Stanford Health Care collaborated with EHR developer Epic (Epic Systems) and Nuance (Microsoft Corporation) to integrate DAX Copilot into clinical documentation work flows. The EHR's mobile app is used to record the physician- patient interaction. The resulting transcript is then processed by an LLM, which generates drafts of 4 note sections that can be accessed via predefined shortcut phrases (SmartSections): history of present illness (HPI), physical exam (PE), results, and assessment and plan (A&P). Physicians insert one or more of these SmartSections into their existing note templates, which then autopopulate once the recording is complete.
Misurac 2024	USA	To assess provider burnout and professional fulfilment associated with Ambient AI technology during a pilot study.	Not reported	University of Iowa Health Care with 38 volunteer physicians and advanced practice providers in ambulatory environments.	Clinical notes and HER.	Ambient AI tool (Nabla Copilot, Nabla, Paris, France), including a mobile device and the AI tool's website. The tool functions by automatically transcribing patient-clinician conversations and utilizing generative AI to create preliminary clinical notes, which were reviewed, edited, and entered into the EHR.
Nguyen 2023	USA	To pilot a digital scribe (DS) in live clinic settings at a National Cancer Institute–designated Comprehensive Cancer Center, evaluate its impact on clinician well-being and documentation burden, and identify implementation facilitators and barriers for effective DS use	Not reported	A National Cancer Institute (NCI) designated Comprehensive Cancer Center in the south eastern UnitedStates. The clinical informatics team received buy-in from 21 clinician champions who participated in this pilot and were invited to complete surveys and interviews about their experiences.	Preliminary draft clinical note for EHR following a clinician-patient encounter.	DS smartphone app, Dragon Ambient eXperience versions 3.0.5, 3.0.6, and 3.0.7 (Nuance: Burlington, MA),28 which could be used in examination rooms to automatically record the visit's discussions. The DS's AI components organized the recorded information into the structure of a visit note, and the vendor's staff completed preliminary editing of the note before releasing the notes to the clinician. Clinicians then review and edit the raw note before signing it. Once signed, the note could be viewed and printed.
Owens 2024	USA	To evaluate the association between ambient voice technology, coupled with natural language processing and AI (DAX™), on primary care provider documentation burden and burnout.	04/2021 - 08/2022	A community teaching health system which consisted of 110 primary care providers at the time of the survey.	Documentation during primary care patient encounters	Ambient voice recognition, coupled with NLP and AI (DAX™). High DAX™ use was expressed as a binary variable dichotomized at >60% of utilization by provider encounters (n=23).
Peine 2023	Germany	To compare paper-based documentation, electronic patient data management systems (PDMSs), and voice information and documentation systems (VIDSs).	02/2021-05/2021	Intensive care units (ICUs). A total of 60 ICU-experienced medical professionals participated in the study (including physicians, medical students after their fourth study year, and ICU nurses).	A fictitious patient was generated using medical data mimicking a typical intensive care patient, including laboratory values, findings, demographic data, and other clinically relevant aspects. The participants were asked to work on 6 different tasks typical in ICU workflow. The first 3 of the tasks were documentation tasks.	Voice information and documentation systems (VIDSs): The AI-based software was installed on a portable system-on-chip computer (NVIDIA AGX Xavier). an adapted version of the Mona system was used, containing: (1) voice handling capabilities: natural language understanding and processing; (2) data processing and preparation algorithms; (3) user interface components; and (4) voice synthesis components. The VIDS interacted with an EHR for each patient. The system was able to extract and display information from the patient data, enter patient care–related tasks, and navigate through charts.

Author (yr)	Country	Objectives	Duration	Setting & healthcare workers	Document & patients	AI-technology & application
Rosenberg 2024	Sweden & Switzerland	To assess both the quality and efficiency of discharge documents generated by ChatGPT-4 in comparison with those produced by physicians.	Not reported	Orthopedic surgery residents at two university hospitals from different European countries (Sweden and Switzerland)	Discharge summary and discharge letter, for 6 fictional orthopedic and trauma cases were written by 2 orthopedic surgery resident	The junior attending orthopedic surgeon and the orthopedic surgery resident who initially generated the discharge documents used a specific prompt for ChatGPT-4 to generate the AI-generated discharge documents. Both physicians were unfamiliar with ChatGPT-4 at the time. This prompt was formulated by 2 authors who are proficient with Chat GPT-4, based on an additional fictional case. This prompt was applied uniformly across all cases,
Shah 2024	USA	To evaluate the pilot implementation of ambient AI scribe technology to assess physician perspectives on usability and the impact on physician burden and burnout.	10/2023 - 01/2024	Stanford Health Care, an academic medical center in Northern California. Physicians were recruited from both faculty and community practice settings across primary care and ambulatory specialties through a combination of purposive and convenience sampling. Exclusion criteria for the pilot included concurrent documentation support with a medical scribe and physicians without access to an Apple smartphone.	Auto-populated draft EHR content.	DAX Copilot ambient AI scribe technology integrated within the EHR, enabling clinicians to record conversations directly from the mobile application. Draft note content was parsed into 4 auto-populated, customizable sections called Epic SmartSections: history of present illness, physical exam, results, and assessment and plan. Once SmartSections were embedded into the EHR note template, AI generated draft content would automatically populate within a few minutes of concluding the recording. Additionally, an attestation SmartSection was embedded into the note template to document that patients had been informed of the recording and that the note had been reviewed by the clinician.
Stults 2025	USA	To understand clinician experience before and after implementing ambient AI.	01/2024 - 07/2024	A large health care organization in Northern and Central California. Clinicians were purposively sampled to be representative of region and specialty; including primary care (58%), medical and surgical subspecialties.	Preliminary draft clinical note for EHR following a clinician-patient encounter.	Ambient artificial intelligence (AI) documentation platform (Abridge AI, Inc.). The platform uses generative AI to listen to a clinical visit and automatically generate a progress note.
Tierney 2024, 2025	USA	Authors build on a prior work and produce novel insights about the sustainability and effectiveness of ambient AI scribes implemented in The Permanente Medical Group following the initial pilot phase.	10/2023 - 12/2024	The Permanente Medical Group (TPMG), a multidisciplinary physician group including about 10,000 physicians working in the Kaiser Permanente Northern California integrated health care delivery system.	Draft clinical notes following clinician-patient encounters	The ambient AI technology uses ML to produce real-time transcripts of clinician–patient encounters to rapidly convert speech collected from microphones on clinicians’ TPMG-provided secure smartphones into text and apply NLP techniques to summarize key clinical content. A change of AI scribe vendor occured during the study period (10/2023 - 12/2024).

Author (yr)	Country	Objectives	Duration	Setting & healthcare workers	Document & patients	AI-technology & application
van-Buchen 2024	Netherlands	To investigate the impact of a commercially available Dutch digital scribe system on clinical documentation efficiency and quality.	Not reported	Leiden University Medical Center consented to participate in the study. 21 medical students with experience in clinical practice and clinical documentation from Leiden University Medical Center consented to participate in our study. All students had a bachelor's degree in medicine and completed a course in clinical documentation.	Clinical summaries after listening to prerecorded mock consultations. Based on 27 recordings of mock consultations between physicians and nonmedical individuals, an internist created 26 vignettes, which delineated a set of symptoms, with a focus on various presentations of chest pain.	Autoscriber is a web-based software application that transcribes and summarizes medical conversations (currently with support for Dutch, English, and German). The pipeline uses a transformer-based speech-to-text model, fine-tuned on proprietary clinical data for transcription and a mixture of large language models such as GPT-3.5 and GPT-4, combined with a tailored prompt structure and additional rules for summarization. The tool also has self-learning functionality, which was not evaluated in this study for practical reasons.
Zuchowski 2022	Germany	This study evaluated the time and cost savings associated with speech recognition technology, and its potential for improving healthcare processes.	Not reported	The nephrology, haematology and emergency departments of Robert Bosch Hospital in Stuttgart, Germany. Clinicians were observed in their normal work environment while they completed their reports following patient consultations.	Preliminary draft clinical note for EHR following a clinician-patient encounter.	Indicda easySpeak (DFC Systems, Aschheim, Germany) and Dragon Naturally Speaking (Nuance Communications, Massachusetts, USA) software.

Supplementar
Detailed data (

Author (yr)	Comparator(s)	Design & sample size	Results	Other notes
Albrecht 2025	Before using the Ambient AI tool.	An anonymous pre- and non-anonymous post-implementation survey evaluated ambulatory clinician perceptions on impact of Abridge. Survey response rates were 51.9% (93/181) pre-implementation and 74.4% (99/133) post-implementation.	Clinician perception of ease of documentation workflow (OR= 6.91, P<.001) and in completing notes associated with usage of the AI tool (OR =4.95, P<.001) was significantly improved. Most respondents agreed that the AI tool decreased documentation burden, decreased the time spent documenting outside clinical hours, reduced burnout risk, and increased job satisfaction, with 48% agreeing that an additional patient could be seen if needed.	Clinician specialty type and number of days using the AI tool did not significantly affect survey responses.
Balloch 2024	The sessions consisted of a 'control' rotation, in which clinicians conducted three consultations using the EHR as they would in practice, and an 'intervention' rotation, in which clinicians conducted three consultations with the AI tool and transferred final notes and clinic letters into the EHR.	Outpatient consultations were simulated with actors and clinicians. Eight experienced clinicians carried out simulated consultations. Forty-seven consultations were performed during the simulation, 23 EHR-alone consultations (excluding one incomplete), and 24 using the AI tool.	AI-produced documentation achieved higher SAIL scores, with consultations 26.3% shorter on average, without impacting patient interaction time. Clinicians reported an enhanced experience and reduced task load.	Clinicians felt less hurried or rushed when using the AI tool compared to the EHR alone.
Barak-Corren 2024	Without assistance of ChatGPT.	A mixed-methods study; 10 pediatric emergency medicine attending physicians, involving 40 (10x4) supervisory notes.	ChatGPT yielded a 40% reduction in time and a 33% decrease in effort for supervisory notes in intricate cases, with no discernible effect on simpler notes. ChatGPT-generated summaries for structured handoffs and family letters were highly rated, ranging from 7.0 to 9.0 out of 10, and most participants favored their inclusion in clinical practice. However, there were several critical reservations, out of which a set of general recommendations for applying ChatGPT to clinical summaries was formulated.	The potential for errors, omissions, and privacy concerns underscores the need for careful, gradual implementation of such tools.
Cao 2024	Pre-DAX documentation time and notes; clinicians' workflow before AI intervention	Clinicians used DAX for documentation after training and completed a nonincentivized survey 30-60 days later. Survey questions were provided by the company for comparison across specialties. 12 clinicians (10 dermatologists and 2 physician assistants) were onboarded and 10 elected to continue using DAX between 02/2021 and 01/2023.	Time spent per day in EMRs decreased from 90.1 to 70.3 minutes (P < .001) post-DAX. DAX users' note contribution percentage decreased by nearly half (P < .001). Note length increased by ~30-50 words (P < .05). 66.7% of clinicians satisfied with documentation turnaround time; 83.3% would be "very disappointed" if DAX was unavailable and felt it "significantly improved" patient experience. Patients reported positive perceptions. Potential cost-savings of ~$13,400 to ~$14,400 annually per clinician compared to in-person scribes at the institution.	Pilot study limited by small sample size and single institution; cost implications noted; AI note quality and comprehensive functionality remain areas for further research.

Author (yr)	Comparator(s)	Design & sample size	Results	Other notes
Duggan 2024	Without using Copilot	A prospective, single-group pre-post quality improvement study. Recruitment identified 46 clinicians, all of whom enrolled in a 2-week implementation and clinician training period followed by 5 weeks of ambient scribe usage.	From baseline to post intervention, use of the ambient scribing tool was associated with 20.4% less time in notes per appointment (from 10.3 to 8.2 minutes; P < .001), 9.3% greater same-day appointment closure (from 66.2% to 72.4%; P <.001), and 30.0% less after-hours work time per workday (from 50.6 to 35.4 minutes per workday; P = .02). SUS scores showed that the ambient scribing tool was easy to use.	According to clinicians' open-ended feedback, ambient scribing–generated notes did not eliminate the burden of clinical documentation. However, ambient scribing decreased the mental effort required for their documentation, both by recording difficult-to-remember details and by eliminating the difficult task of writing a note completely from scratch. The need for substantial editing and proofreading of the AI-generated notes sometimes offset the time saved
Galloway 2024	Before using the Ambient AI tool.	BAC: A voluntary web-based survey to a convenience sample of 117 clinicians, at the time of pilot onboarding and 60 days after. 31 participants completed both the onboarding and follow-up surveys.	Concerning the ease of their current documentation process, 32.3% responded positively compared to 48.4% post-intervention (p = 0.023). When asked about the impact of their current documentation process on their well-being, 71% responded negatively compared to 38.7% post-intervention (p = 0.010). When asked about the impact of their current documentation process on the patient experience, 35.5% responded negatively compared to 6.5% post-intervention (p = 0.005).	Limitations to this study include small sample sizes and a questionnaire change between the pre- and post-interven tion surveys, which may have impacted how some questions were interpreted.
Garcia 2024	Pre-survey vs. post-survey. The pilot period lasted for 35 days extending from 10/07/203 through 13/08/2023. A corresponding 35-day prepilot period extending from 29/05/2023 through 02/07/2023, was used for comparison.	BAC: A 5-week, prospective, single-group quality improvement study. Of the 197 clinicians enrolled in the pilot, 162 were included in the study analysis. The survey group consisted of 73 participants (45.1%) who completed both the presurvey and postsurvey.	The mean AI-generated draft response utilization rate across clinicians was 20%. There was no change in reply action time, write time, or read time between the prepilot and pilot periods. There were statistically significant reductions in the 4-item physician task load score derivative (mean [SD], 61.31 [17.23] presurvey vs 47.26 [17.11] postsurvey; paired difference, −13.87; 95% CI, −17.38 to −9.50; P<.001) and work exhaustion scores (mean [SD], 1.95 [0.79] presurvey vs 1.62 [0.68] postsurvey; paired difference, −0.33; 95% CI, −0.50 to −0.17; P<.001).	No changes in overall reply time, read time, or write time were found. It may be that switching from writing to editing may be less cognitively taxing despite taking the same amount of time.
Haberle 2024	A peer-matched control cohort	A peer-matched controlled cohort study. A total of 99 providers representing 12 specialties enrolled in the study; 76 matched control group providers were included for analysis.	Median utilization of DAX was 47% among active participants. We found positive trends in provider engagement, while non-participants saw worsening engagement and no practical change in productivity. There was a statistically significant worsening of after-hours EHR. There was no quantifiable effect on patient safety.	The first published study describing the use and practical outcomes of ambient listening AI documentation in real-world outpatient clinical care—and at modest scale.
Hudson 2025	Usual note writing process	Participants were assigned with 2 comparable clinic sessions, and were randomized to first use either Abridge (an EHR-integrated ambient AI platform) or usual note writing process. Then they switched to the alternate method during the second session. 40 ambulatory providers at a large academic institution	Use of Abridge was associated with both a significantly lower NASA-TLX composite score -mean difference of 60.7% (221.20 vs 118.20) - and a reduction in each individual subdimension of effort (60.3%, 75.25 vs 40.38), mental demand (64.6%, 74.00 vs 37.88), and temporal demand (57.1%, 72.00 vs 40.00) (all with P<.001).	

Author (yr)	Comparator(s)	Design & sample size	Results	Other notes
Janota 2024	3 participants received Case 1 and 3 received Case 2, and they were asked to write standardized discharge reports and record the time required.	NRS: Quality of the 8 reports, including 2 AI-generated, was assessed by 4 attending physicians who did not know who wrote each discharge summary.	Discharge summaries generated by AI are more efficient than discharge summaries prepared by clinic staff. The AI was particularly effective in terms of coherence and information structure.	For physicians of foreign origins, the correct grammatical, orthographical and clinical composition of a discharge summary can be particularly challenging.
Kaufman 2016	Standard (S) vs NLP (N). Two documentation parts: (1) History & physical exam (S or N), and (2) assessment & plan (S or N): SS, SN, NS, NN.	Compared MediSapien NLP for structured data capture vs. a standard structured data capture protocol, as well as 2 novel hybrid protocols (NLP-Standard and Standard-NLP). There were 31 participants, and a total of 118 notes were documented across the 3 subject areas.	The NLP-NLP protocol required a median of 5.2 - 8.5 minutes compared with 16.9 -21.2 minutes using the Standard-Standard protocol, and 13.8 -21.3 minutes using the Standard-NLP protocol. Using 8 out of 9 characteristics measured by the PDQI-9 instrument, the NLP-NLP protocol received a median quality score sum of 24.5; the Standard-Standard protocol received a median sum of 29; and the Standard-NLP protocol received a median sum of 29.5. The mean total score of the usability measure was 36.7 when the participants used the NLP-NLP protocol compared with 30.3 when they used the Standard-Standard protocol.	
Liu (1, 2) 2024	Without using the AI tool.	NRS: the intervention group clinicians (n = 112) vs a control group of clinicians without using the AI-tool (n = 117). The survey was anonymously emailed to 230 participants before and 5 weeks after the intervention implementation; 85 and 55 responded, respectively, in the AI and control group.	In the intervention group, 40 of 85 (47.1%) reported decreased time on the EHR at home vs. 8 of 55 (14.5%) in the control group (P < .001); and 38 of 85 (44.7%) reported decreased weekly time on the EHR outside normal work hours vs. 11 of 55 (20.0%) in the control group (P = .003). Moreover, 37 of 85 intervention respondents (43.5%) reported decreased time on documentation after the visits vs. 1 0of 55 (18.2%) in the controlgroup (P = .002) and 38 of 85 (44.7%) reported less frustration using the EHR vs. 8 of 55 (14.5%) in the control group (P <.001).	(1) A research letter, also included a study by the same team. (2) No definition given regarding "mean ratio" in the paper. Mentioned that "widespread implementation of DAX in its current form is unlikely to generate appreciable gains for health care systems looking to increase productivity."

Author (yr)	Comparator(s)	Design & sample size	Results	Other notes
Ma 2024	Outcomes from the pilot period were compared against a 2-month baseline pre-pilot period.	BAC: A prospective quality improvement study at a large academic medical center with 45 physicians from 8 ambulatory disciplines over 3 months.	The ambient AI scribe was utilized in 9629 of 17 428 encounters (55.25%) with significant interuser heterogeneity. Compared to baseline, median time per note reduced significantly by 0.57 minutes. Median daily documentation, afterhours, and total EHR time also decreased significantly by 6.89, 5.17, and 19.95 minutes/day, respectively.	There was notable individual variability in how physicians used the tool, and in time savings. This heterogeneity implies that certain user phenotypes may derive more utility from current ambient AI technology than others.
Misurac 2024	Without using the Ambient AI tool.	A 5-week pre-post observational study . Pre-test and post-test surveys were completed by 35/38 participants (92% survey completion rate).	Median burnout score improved from 4.16 to 3.16 (p=0.005). With validated Stanford PFI cutoff for overall burnout 3.33, burnout rates decreased from 69% to 43%. There was a notable improvement in interpersonal disengagement scores (3.6 vs. 2.5, p<0.001), although work exhaustion scores did not significantly change. Professional fulfillment showed a modest, non-significant increase (6.1 vs. 6.5, p=0.10).	Clinicians used a mobile device and separate website to use the technology, so that comprehensive integration of the tool into EHR is not required in this case.
Nguyen 2023	Without using the AI tool.	We conducted a survey that assessed demographics, burnout, work related stressors, and sleep quality at baseline and 1 month after using the DS. Of the 21 participants who were sent the baseline survey, 10 (47.6%) responded. Nine also responded to the 1-month survey. Eight participants completed the interview.	Across nine survey responses and eight interviews, we found that although feasibility scores were slightly lower than our cutoff point (15.2 v 16.0), clinicians rated the DS as marginally acceptable (16.0) and appropriate (16.3). Usability was considered marginally usable (68.6 v 68.0). Although the DS did not significantly improve burnout (3.6 v 3.9, P = .081), it improved perceptions of having sufficient documentation time (2.1 v 3.6, P = .005).	Clinicians reported varying amounts of time needed before they felt comfortable using the DS. Although some needed a month before feeling comfortable, one clinician reported feeling in stantly comfortable and another reported never feeling comfortable. We used complete case analysis to address missing data.
Owens 2024	Low DAX users (n=60)	A cross sectional cohort survey to evaluate provider burnout and a retrospective before and after subgroup to evaluate documentation burden. 83 participants completed the survey; 19 with high implementation provided data in the analysis of documentation burden (time, percent contribution, and length).	High DAX™ use was associated with significantly less burnout on the OLBI disengagement sub-score (MD -2.1; 95% CI -3.8 to -0.4) but not the OLBI disengagement sub-score (-1.0; -2.9 to 1.0) or total score (MD -3.0; -6.4 to 0.3). After DAX™ implementation average documentation time in notes per encounter was significantly reduced by 28.8% (1.8 min; 1.4 to 2.2).	The only variable associated with total and sub-scores on the OLBI was FTE. However, we found no significant change in the estimates in OLBI scores after adjusting for FTE.
Peine 2023	Traditional, paper-based documentation systems compared with PDMSs and newer AI-based VIDSs.	The crossover design was chosen to ensure the comparability of all 3 interventions with respect to confounding variables. The participants were asked to work on 6 different tasks typical in ICU workflow. The first 3 of the tasks were documentation tasks, and the order in which the participants were presented with the different study arms was randomized. *A total of 60 ICU-experienced medical professionals.	The tasks were completed significantly faster with the VIDS than with the PDMS (Cohen d=1.61; P<.001) or paper documentation (Cohen d=2.63; P<.001). Significantly fewer errors were made with VIDS than with the PDMS (Cohen d=0.45; P=.03) and paper-based documentation (Cohen d=1.45; P<.001). The analysis of the mental workload of VIDS and PDMS showed no statistically significant difference (P=.06). However, the analysis of subjective user perception showed a statistically significant perceived benefit of the VIDS compared to the PDMS (P<.001) and paper documentation (P<.001).	A single fabricated patient but different documentation tasks: eg, "Document 300 mg amiodarone IV now"; "Document 1.5 g piperacillin/tazobactam (Tazobac) intravenously now/at 10:00 AM"; "Document the administration of a red blood cell concentrate/fresh frozen plasma at 1:00 AM for procedures numbered 1101002233 and indication active bleeding"; "Document 20 mg furosemide now"

Author (yr)	Comparator(s)	Design & sample size	Results	Other notes
Rosenberg 2024	A junior attending orthopedic surgeon and a senior orthopedic surgery resident independently created 2 discharge documents for 3 cases each: a discharge summary and a discharge letter	Both human and GPT-4-generated documents were assessed by an expert panel of orthopedic residents and surgeons (n=15), blinded to the author of the document. 6 cases with 12 documents in total	Overall, both ChatGPT-4 and physician generated notes were comparable in quality. Notably, Chat GPT-4 generated discharge documents 10 times faster than the traditional method. 4 events of hallucinations were found in the ChatGPT-4-generated content, compared with 6 events in the human/physician produced notes.	The ChatGPT-4-generated notes were subjected to evaluation without prior human review, so that the reduction in time was over-estimated in this study. The review and approval of ChatGPT-4-generated notes may be more time-consuming than assessing notes written by physicians.
Shah 2024	Burden and burnout scores were collected in both pre and post surveys.	BAC: This prospective quality improvement study was conducted at Stanford Health Care with 48 physicians over a 3-month period. Of the 48 physicians who remained enrolled in the pilot (median use of 12.8 weeks), 38 were included in the paired pre and post-survey analysis and 46 in the unpaired post- survey analysis.	Paired survey analysis (n=38) revealed large statistically significant reductions in task load (−24.42, p <.001) and burnout (−1.94, p <.001), and moderate statistically significant improvements in usability scores (+10.9, p <.001). Post-survey responses (n=46) indicated favorable utility with improved perceptions of efficiency, documentation quality, and ease of use.	Irrespective of time savings, clinicians may find other benefits, such as improved efficiency, documentation quality, and physician-patient engagement. Several physicians self-reported increased documentation time, this may have been due to an initial learning curve, early hypervigilance, or repurposing time spent writing into time spent editing.
Stults 2025	Before and after implementing ambient AI.	This quality improvement study was a pilot evaluation with before and after survey and EHR metrics conducted at a large health care organization in Northern and Central California. Among 100 clinicians, 92 clinicians had EHR metrics, and 57 completed both preimplementation and postimplementation surveys.	Following AI implementation, clinicians showed a significant reduction in notes per appointment from 6.2 to 5.3 minutes, with a larger decrease among female clinicians; there was no significant change in off-hour EHR activities. The proportion of clinicians spending ≤1 hour/week on notes outside clinic hours increased from 14% to 54.4%. Mental demand, hurried pace, and effort scores on NASA-TLX significantly decreased, indicating reduced workload, while burnout showed a non-significant decline. Clinicians reported improved ability to give undivided patient attention (93% post vs. 58% pre) and increased documentation length. Additionally, 72% agreed that Abridge improved work satisfaction, especially among primary care providers (86%) compared to other specialties.	Available subgroup analysis results by specialties.<br>While our clinicians had a significant decrease in time spent in notes per appointment, they did not see an association with off-hour EHR activities or other after-hours work. Interestingly, progress notes and documentation length were longer after use of ambient AI, which is similar to previous findings. Ambient AI has since been fully integrated into the Sutter EHR, and we are in the process of collecting data to understand experiences of clinicians since this change.
Tierney 2024, 2025	Pre-implementation of the Ambient AI tool, and users vs nonusers.	Difference-in-difference analysis: changes before and after AI use among AI scribe users, vs. nonusers, and adjusted for clinician, specialty, and appointment volume. During the >1 year since implementation, of the 10,000 physicians and staff provided access to accounts, 7260 TPMG physicians enabled the tool in a total of 2,576,627 encounters across a wide array of medical specialties and locations.	AI scribes continue to demonstrate efficacy in reducing physician workload, in aggregate producing estimated time savings in documentation of more than 15,700 hours for users — equivalent to 1,794 working days — compared with nonusers, over 1 year of use.	A longer follow-up of Tierney 2024. Physicians have had an overwhelmingly positive experience with ambient AI scribes, largely due to the impact on time savings and cognitive load, with the AI scribes helping them to remember details of conversations. Patients also reported a generally positive impact of AI scribes on their care experiences.

Author (yr)	Comparator(s)	Design & sample size	Results	Other notes
van-Buchen 2024	The consultations were summarized using 3 methods: manual summaries, fully automated summaries, and automated summaries with manual editing.	NRS: 21 medical students with experience in clinical practice and clinical documentation from Leiden University Medical Center consented to participate in our study. All students summarized 4 consultations manually, then 8 consultations using Autoscriber, and finally 4 consultations manually to minimize a learning effect. In total, we collected 156 manual summaries, 137 automatic summaries, and 137 edited summaries from 21 students.	The median time for manual summarization was 202 seconds against 186 seconds for editing an automatic summary. Without editing, the automatic summaries attained a poorer PDQI-9 score than manual summaries (P<.001). Automatic summaries were found to have higher word counts but lower lexical diversity than manual summaries (P<.001). The study revealed variable impacts on PDQI-9 scores and summarization time across individuals.	Digital scribes may be more beneficial to some physicians than to others and could play a role in improving the reusability of clinical documentation. During editing, medical students most often added context and details, while removing overly general statements and irrelevant text.
Zuchowski 2022	Without using SR tool.	NRS: a prospective, non-randomised design. Clinicians were directly observed while completing medical documentation. In total, 15 clinicians participated over a study period of 6 months, producing 313 samples, of which 163 used speech recognition software and the remaining 150 used typing. A survey was also completed by 31 clinicians to gauge their level of acceptance of speech recognition software for medical documentation.	On average, medical documentation using speech recognition software took just 5.11 minutes to complete the form, compared to 8.9 minutes typing, representing significant time savings. The error rate was also found to be lower for speech recognition software. However, 55% of clinicians surveyed stated that they would prefer to type their notes rather than use speech recognition software and perceived the error rate of this software to be higher than typing.	As speech recognition technology can take some time to get used to, only clinicians who habitually used either this method or typing for medical note taking were selected. The use of direct human observation in a clinical setting to evaluate speech recognition software for medical documentation is unique to the present study.
